# Editorial: New insights into caring for pediatric patients with cystic fibrosis

**DOI:** 10.3389/fped.2023.1243496

**Published:** 2023-08-09

**Authors:** Stephanie Bui, Laurence Delhaes, Gael Dournes, Philippe Reix, Michael John Fayon

**Affiliations:** ^1^ Paediatric Cystic Fibrosis Reference Center (CRCM), Bordeaux University Hospital, Hôpital Pellegrin-Enfants, Bordeaux, France; ^2^ Centre de Recherche Cardio-Thoracique de Bordeaux, U1045, CIC 1401, Bordeaux University Hospital, Bordeaux, France; ^3^ Service de Parasitologie-Mycologie, UMR 12 19, U1045, Bordeaux University Hospital, Bordeaux, France; ^4^ Service d’Imagerie Thoracique et Cardiovasculaire, Bordeaux University Hospital, Bordeaux, France; ^5^ Lyon University Hospital, Hospices Civils de Lyon, Lyon, France; ^6^ Paediatric Cystic Fibrosis Reference Center (CRCM), UMR 5558, Centre National de Recherche Scientifique (CNRS), Lyon, France

**Keywords:** cystic fibrosis, standard of care, modulators, telehealth, artificial inteligence, nutritional status, newborn screening (NBS)

**Editorial on the Research Topic** New insights into caring for pediatric patients with cystic fibrosis

Cystic fibrosis (CF) is a rare and severe inherited autosomal recessive disease with more than 2,000 mutations identified in registries around the world. CF is related to mutations in the CF transmembrane conductance regulator (CFTR) gene, which encodes the CFTR epithelial ion channel involved in chloride and bicarbonate transport leading to impaired mucus hydration and clearance (1–3). The reduced functional protein at the cell surface is characterised by multiple exocrinopathies including lung involvement due to thick dehydrated bronchial secretions, favouring chronic and acute infections and inflammation (1). Pulmonary exacerbations occur frequently and are associated with poor nutritional status, leading to progressive lung function decline and death in people with CF (pwCF) (4, 5). To prevent such morbidity and mortality, standards of care rely on multidisciplinary team management and trimonthly follow-up (6–8).

The present special issue on CF focuses on new insights into CF care. One review shows that although neonatal screening is set up in most countries, physicians should be aware that CF should also be evoked based on non-specific clinical signs.

Furthermore, until recently, for most pwCF, management relied mainly on symptomatic therapies (physiotherapy, mucolytics, and pancreatic enzymes, etc.) (6, 8). Such treatments have shown their ability to slow down the progression of CF and enhance life expectancy (9). In the last few years, new therapeutic approaches for treating CF have been developed aiming at restoring CFTR function in a wider proportion of pwCF, notably those bearing at least one F508del CFTR mutation (around 70% to 80% of pwCF). F508del mutation is responsible for processing and trafficking defects in the mutated F508del CFTR protein, leading to its degradation before it reaches the epithelial cell surface (1–3). Modulators generally associate one, two, or three CFTR correctors, which enhance the cellular protein processing and trafficking, and a potentiator, which increases the channel-opening probability (1, 4). At the present time, triple combination therapy associating two correctors (elexacaftor and tezacaftor) and a potentiator (ivacaftor) is available for a large proportion of pwCF upwards of 6 years of age. These new molecules have revolutionised CF prognosis, decreased treatment burden, and improved daily quality of life (10, 11).

This issue also includes a study on the evolution of nutritional intake in children with CF (cwCF), in particular after administration of the first class of modulators [lumacaftor–ivacaftor (LI)] in F508del homozygous patients. Regarding the pulmonary outcome, which is slightly improved under LI (12–14), assessments now include artificial intelligence to help in deciphering respiratory imaging structural changes.

Finally, regarding the real-life cohorts for the most recent modulator (elexacaftor–tezacaftor–ivacaftor: ETI), who have demonstrated dramatic improvement in their lung function and nutritional status (10, 11), we include a paper describing the validity and feasibility of telehealth home monitoring with spirometric connected devices to help multidisciplinary teams follow up the disease in this new therapeutic era.

Although we have experienced dramatic improvements in the diagnosis, management, and follow-up of CF, the next steps will be to diagnose and treat all patients with a personalised medicine approach, including those without mutations currently eligible for modulator therapies. Such therapies should also be made available to more and more pwCF throughout the world.
